# Insights from LC-MS-based cerebrospinal fluid metabolomics in tuberculous meningitis

**DOI:** 10.3389/fmolb.2026.1861630

**Published:** 2026-06-17

**Authors:** Ontefetse Neo Plaatjie, A. Marceline Tutu van Furth, Martijn van der Kuip, Shayne Mason

**Affiliations:** 1 Department of Biochemistry, Biomedical and Molecular Metabolism (BioMMet), Faculty of Natural and Agricultural Sciences, North-West University, Potchefstroom, South Africa; 2 Department of Paediatric Infectious Diseases and Immunology, Emma Children’s Hospital, Amsterdam University Medical Center, Amsterdam, Netherlands

**Keywords:** biomarker, cerebrospinal fluid (CSF), LC-MS/MS, metabolism, metabolomics, tuberculous meningitis (TBM)

## Abstract

Tuberculous meningitis (TBM) remains the most devastating form of extra-pulmonary tuberculosis (TB) and is associated with high mortality and neurological deficits, often due to delayed diagnosis. Disease outcome in TBM depends critically on early diagnosis and timely initiation of treatment. However, the non-specific clinical presentation of TBM poses a major diagnostic challenge, as no single test can reliably establish a definitive diagnosis. The recommended diagnostic approach, GeneXpert MTB/RIF ultra, combined with mycobacterial culture of cerebrospinal fluid (CSF), remains limited by suboptimal sensitivity and restricted availability in many resource-constrained settings. These limitations underscore the urgent need for novel biomarkers that reflect TBM-specific pathophysiology and enable a single rapid, reliable, and accessible diagnosis. This perspective paper draws on insights from LC-MS-based CSF metabolomic profiling to highlight metabolic alterations in TBM. Key metabolic pathways–fatty acid β-oxidation reflected by altered acylcarnitine profiles, amino acid perturbations, and the tryptophan-kynurenine pathway–are discussed in relation to cellular energy disruption and neuroinflammation. Based on these alterations, free carnitine and quinolinic acid emerge as priority candidates for further investigation. Free carnitine reflects TBM-associated energy dysregulation, while quinolinic acid appears to mirror the severity of neuroinflammation. Future studies should focus on validating these metabolites in independent cohorts and on assessing their translational potential in more readily accessible biofluids, with the ultimate goal of enabling simplified, widely implemented diagnostic assays.

## Introduction

Tuberculous meningitis (TBM) is a severe form of tuberculosis (TB) and is frequently fatal if left untreated. It is characterized by leptomeningeal inflammation caused by infection with *Mycobacterium tuberculosis,* the pathogen responsible for pulmonary TB. Although TBM accounts for approximately 1% of all TB cases worldwide, its clinical impact is disproportionally high (Méchaï and Bouchaud, 2019). In 2024, an estimated 10.7 million individuals developed TB globally (World Health Organisation, 2025)^,^ suggesting that approximately 107,000 cases of TBM occurred during this period. Young children, particularly those aged 2–5 years, and people living with HIV are at increased risk of developing TBM due to immature or compromised immune function (Méchaï and Bouchaud, 2019). While TBM is potentially curable with early treatment, diagnosis is often delayed, leading to presentation at advanced stages of the disease, where outcomes are poor, and survivors often sustain permanent neurological deficits.

Tuberculous meningitis is treatable when diagnosed promptly, and treatment is initiated before progression to severe neurological complications (Thao et al., 2018; Donovan et al., 2025). Current TBM treatment strategies are adapted from pulmonary TB regimens. The World Health Organization (WHO) recommends an initial 2-month intensive phase consisting of isoniazid, rifampicin, ethambutol, and pyrazinamide (HRZE), followed by a continuation phase of isoniazid and rifampicin for an additional 10 months in children with confirmed TBM. More recently, the WHO conditionally recommended a shorter 6-month intensive regimen for children without suspected or confirmed multidrug-resistant/rifampicin-resistant TB (MDR/RR-TB), comprising higher-dose isoniazid and rifampicin, pyrazinamide, and ethionamide in place of ethambutol (Donovan et al., 2025; van Toorn et al., 2014). Although challenges such as multidrug resistance and prolonged treatment duration remain, TBM is curable when identified early. The high mortality associated with the disease is largely attributable to delayed diagnosis and late initiation of therapy.

The early diagnosis of TBM is challenging because the initial clinical presentation often overlaps with that of other forms of meningitis, even though it requires pathogen-specific treatment. Patients commonly present with nonspecific symptoms, including headache, low-grade fever, vomiting, and neck stiffness. Cerebrospinal fluid (CSF) findings in TBM typically include low glucose (≤2.22 mmol/L), elevated protein (>1.0 g/L), and pleocytosis (>20 cells/µL) with lymphocyte predominance (>50%) (Marais et al., 2010; Solomons et al., 2015). However, these parameters, either individually or in combination, lack sufficient specificity to establish a definitive diagnosis. Mycobacterial culture remains the diagnostic gold standard but is limited by prolonged turnaround times, often delaying treatment initiation. Molecular assays such as GeneXpert *Mycobacterium Tuberculosis*/Rifampicin (MTB/RIF) Ultra have therefore been recommended as first-line diagnostic tools; nevertheless, their sensitivity remains imperfect, and access to these tests is limited in many resource-constrained settings. Consequently, there is a critical need for a rapid, sensitive, and accessible diagnostic approach that enables early differentiation of TBM from other forms of meningitis, thereby improving clinical outcomes and reducing mortality.

Identifying TBM-specific biomarkers has the potential to substantially improve current diagnostic challenges. Metabolomics, the systematic analysis of small-molecule metabolites that represent the end products of cellular processes, has increasingly been applied to the study of TBM. Untargeted metabolomics studies have revealed significant perturbations across multiple metabolic pathways in TBM (Mason and Solomons, 2021), prompting the use of targeted approaches to interrogate specific metabolites with potential diagnostic relevance. Liquid chromatography-mass spectrometry (LC-MS) is particularly well suited for targeted metabolomic analysis due to its high sensitivity and the ability to accurately quantify low-abundance metabolites. This is especially relevant in CSF, where metabolite concentrations are typically low but reflect disease-associated biochemical changes within the central nervous system (CNS). Given the close physiological relationship between CSF and the CNS, CSF metabolomics represents a promising platform for discovering TBM-associated diagnostic biomarkers. In this perspective paper, we discuss metabolic insights gained from LC-MS analysis of CSF in TBM, with a particular focus on candidate metabolites with potential utility for differential diagnosis and their translational implications.

## Acylcarnitines and disrupted energy metabolism

Targeted LC-MS analysis of CSF acylcarnitines in pediatric TBM revealed significant elevations of free carnitine and short-chain acylcarnitines (carnitine derivatives), with free carnitine showing the most pronounced increase and a large effect size in differentiating TBM from non-meningitis controls (NMC) and viral meningitis (VM) (Plaatjie et al., 2025a). Importantly, the concentrations of L-carnitine and acetylcarnitine observed in TBM cases were substantially higher than established pediatric CSF reference ranges, previously defined in a non-TBM pediatric population (Plaatjie et al., 2025b). L-carnitine is an endogenous compound found in mammalian species and plays a critical role in energy production. Carnitines are widely known for their central role in mitochondrial fatty acid transport, particularly long-chain fatty acids, from the cytosol into the mitochondria for β-oxidation (Dambrova et al., 2022; Plaatjie et al., 2024); a pathway increasingly relied upon during states of high energy demand and limited glucose availability in the body. In TBM, profound neuroinflammation and immune activation markedly increase cerebral energy demands and may contribute to altered brain metabolism. Glucose, the brain’s primary energy source, is reduced in TBM, particularly in CSF. Under these conditions, the brain may partially rely on alternative substrates, including ketone bodies derived from hepatic β-oxidation (Dai et al., 2017; Jensen et al., 2020), a process dependent on efficient carnitine-mediated fatty acid transport.

The observed elevation of free carnitine and short-chain acylcarnitines in TBM may therefore reflect altered mitochondrial flux and metabolic stress within the CNS, potentially indicating increased reliance on fatty acid-derived energy or incomplete β-oxidation during sustained inflammation. Short-chain acylcarnitines, particularly acetylcarnitine, are commonly linked to acetyl-CoA overflow and buffering during periods of high metabolic demand and may also reflect mitochondrial dysfunction, collectively indicating disrupted energy homeostasis in TBM (Schroeder et al., 2012). A CSF metabolomic study in adults with TBM similarly reported elevated levels of multiple carnitines, which were associated with concurrent activation of mitochondrial beta-oxidation of fatty acids (Tomalka et al., 2024). In children with TBM, a ^1^H NMR metabolomics study identified elevated carnitine as an important metabolite (Van Zyl et al., 2020). Collectively, these studies underscore the importance of carnitines in TBM. However, carnitine alterations were not observed in VM (Plaatjie et al., 2025a), which aligns with the preserved glucose availability in this condition and underscores the specificity of carnitine metabolic changes to TBM.

Further supporting its specificity for TBM, free carnitine showed positive correlations with basal meningeal enhancement and hydrocephalus, key radiological features of TBM. Acetylcarnitine and propionylcarnitine also correlated with CSF leukocyte and lymphocyte counts, linking alterations in energy metabolism to the heightened immune response characteristic of TBM. However, these short-chain acylcarnitines alone showed limited ability to discriminate TBM from VM, likely because elevated CSF white blood cell counts are a shared feature of both conditions. This overlap in inflammatory response may therefore explain their reduced specificity in differentiating between the two diseases. However, their combined evaluation with free carnitine, which correlated with TBM-specific features, enhanced diagnostic performance. Thus, these findings highlight free carnitine as a biologically informative marker of metabolic stress in TBM, and, when combined with its derivatives–acetylcarnitine and propionylcarnitine–they could serve as informative markers of both metabolic stress and inflammation.

### Tryptophan-kynurenine and neuroinflammation

Analysis of CSF tryptophan and its downstream metabolites in pediatric TBM revealed marked disruptions in tryptophan metabolism, particularly along the tryptophan-kynurenine pathway. CSF tryptophan levels were significantly reduced in both TBM and VM, with no significant difference between the two conditions (Plaatjie et al., 2025c). Tryptophan is an essential amino acid, and approximately 95% of its metabolism occurs *via* the indoleamine 2,3-dioxygenase (IDO) pathway into the kynurenine pathway, generating a range of biologically active metabolites with diverse functions, including immunomodulatory, neuroactive, antioxidant, neurotoxic, and neuroprotective effects (Tanaka et al., 2021; Yan et al., 2023; Guo et al., 2024). Increasing evidence indicates that tryptophan metabolism, especially *via* the kynurenine pathway, is a key interface between immune activation and neuroinflammation (Huang et al., 2023; Kondo et al., 2024). In addition to reduced CSF tryptophan in both TBM and VM, our study identified a negative correlation between CSF tryptophan and lymphocyte counts, a hallmark of neuroinflammation common to both conditions. This association likely explains the comparable depletion of tryptophan observed in TBM and VM and is consistent with the limited diagnostic performance of tryptophan in distinguishing between the two diseases. Collectively, these findings suggest that the clinical relevance of tryptophan lies in its reflection of immune activation rather than disease specificity. This interpretation is further supported by adult TBM studies reporting reduced CSF tryptophan levels (van Laarhoven et al., 2018) and an inverse association between tryptophan and interferon-γ (IFN-γ), a key mediator of the inflammatory immune response (Ardiansyah et al., 2023).

Although tryptophan itself lacked disease specificity, metabolites downstream of the kynurenine pathway provided additional insight into neuroinflammatory and neurotoxic processes in TBM. Kynurenine was elevated in TBM; however, quinolinic acid was the most markedly increased downstream metabolite (Plaatjie et al., 2025c). Pathway activity was assessed using the kynurenine/tryptophan ratio as a surrogate marker of IDO activation and the quinolinic acid/tryptophan ratio as an indicator of downstream pathway flux. The kynurenine/tryptophan ratio increased modestly in both TBM and VM, consistent with generalized immune-driven tryptophan catabolism. In contrast, the quinolinic acid/tryptophan ratio was markedly elevated in TBM compared with both VM and NMC, a pattern not observed between VM and controls.

Clinically, this profile suggests that although tryptophan catabolism is activated in meningitis (i.e., this pathway is associated with inflammation), TBM is characterized by preferential shunting of kynurenine toward downstream metabolites. Quinolinic acid, a well-established neurotoxin and mediator of neuroinflammation, may therefore reflect disease-specific amplification of immune and metabolic pathways in TBM. Supporting this interpretation, quinolinic acid showed positive correlations with basal enhancement and hydrocephalus, hallmark features of TBM, indicating an association with disease severity and structural CNS involvement. Furthermore, quinolinic acid showed strong discrimination between TBM and both VM and NMC, but lacked discrimination between VM and NMC, thereby supporting its specificity for TBM-related pathophysiology. Importantly, the relevance of quinolinic acid is further supported by an independent NMR-based study in pediatric TBM, which identified it as a potential diagnostic marker in urine (Isaiah et al., 2024), suggesting that its diagnostic utility may extend beyond CSF.

### Amino acids alterations

Analysis of CSF amino acids (AAs) revealed a generalized increase in AAs levels in TBM compared with both NMC and VM (Plaatjie et al., 2026). Among these, lysine, glycine, alanine, tyrosine, methionine, and arginine showed the strongest discriminatory effects, highlighting widespread metabolic perturbations associated with TBM. AAs serve as central metabolic intermediates in the CNS, contributing to neurotransmission, immune regulation, redox balance, and energy metabolism. Their elevation in TBM therefore suggests substantial disruption of cerebral metabolic homeostasis.

One potential explanation for increased AA levels is enhanced protein breakdown in TBM; however, the lack of a significant correlation between AA and CSF protein levels argues against nonspecific protein leakage as the primary source. Instead, these alterations may reflect adaptive metabolic responses to sustained energy stress. Under conditions of high energy demand, AAs can be mobilized to support energy production *via* oxidative pathways or anaplerotic flux into the tricarboxylic acid (TCA) cycle (Chandel, 2021; White, 2015). These observed elevations in AAs may therefore represent a compensatory response to increased energy demand and inflammatory stress within the CNS. The findings of this study are consistent with previous reports of elevated AAs in TBM, with alanine, glycine, valine, lysine, proline, and tyrosine showing reproducible elevations in both pediatric and adult TBM cohorts (Van Zyl et al., 2020; Binici et al., 2023; Mason et al., 2017).

Among individual AAs, glycine showed a positive association with inflammatory markers, correlating with CSF leukocyte and lymphocyte counts (Plaatjie et al., 2026), suggesting a link to immune activation and persistent neuroinflammation in TBM, despite glycine’s anti-inflammatory and immunomodulatory properties (Aguayo-Cerón et al., 2023). The ability to differentiate TBM from VM was limited, likely because it reflected a generalized inflammatory response, a feature shared by both TBM and VM, rather than a TBM-specific feature. Although their diagnostic utility in isolation is limited, AA alterations may provide important mechanistic insights into host immune responses and could enhance diagnostic performance when combined with more TBM-specific metabolites.

## Discussion

The overarching goal of biomarker discovery in TBM is to develop rapid, accessible, and reliable diagnostic tools that enable early detection and timely treatment. The metabolic alterations identified in this study provide foundational insights by highlighting candidate metabolites that may differentiate TBM from other forms of meningitis. Although lumbar punctures are invasive, CSF remains the most biologically relevant matrix for biomarker discovery because of its close anatomical and physiological connection to the CNS and its ability to reflect disease-specific pathology. Therefore, CSF-based metabolomic profiling is a critical first step that can yield robustly validated biomarkers, which can then be assessed in more accessible biofluids such as blood or urine, paving the way for simplified diagnostic assays that could be widely implemented.

Several CSF parameters are routinely used in the clinical evaluation of suspected TBM, including reduced glucose, elevated protein, increased lactate, and lymphocytic pleocytosis. While these markers are useful for raising clinical suspicion, they lack sufficient specificity to reliably distinguish TBM from other forms of meningitis. The substantial overlap in these conventional CSF profiles contributes to diagnostic uncertainty and delays in treatment initiation. In this context, metabolomic profiling offers an opportunity to move beyond general inflammatory markers toward disease-associated metabolic signatures that reflect the underlying pathophysiological processes specific to TBM.

The findings highlighted in this perspective underscore the potential of LC-MS CSF metabolomics to inform the diagnosis and biological understanding of TBM. Among the observed metabolic alterations, free carnitine and quinolinic acid emerged as the most promising candidates for clinical translation, showing disease specificity and strong associations with key TBM-related features. Altered amino acids should also be considered, as they are consistently elevated in TBM. Future studies should prioritize validating these metabolites in larger, independent cohorts, including comparisons with other forms of bacterial meningitis, to more clearly define their specificity for TBM. Further investigation across well-characterized disease stages will be essential to determine whether free carnitine and quinolinic acid are elevated early in the disease course or predominantly reflect advanced neuroinflammation. Such an investigation may also reveal whether these metabolites can track disease progression and treatment response, thereby extending their application beyond diagnosis to serve as prognostic and monitoring tools. In addition, incorporating kynurenic acid measurements into future studies would enable assessment of the balance between neurotoxic and neuroprotective metabolites of the kynurenine pathway, providing further mechanistic insights into TBM-associated neuroinflammation. Further investigation of additional metabolic pathways, particularly lipid and fatty acid metabolism, is warranted, as these pathways remain relatively underexplored in TBM, despite evidence of altered lipid metabolites in untargeted studies and the brain’s high lipid content.

Given the biological complexity of TBM, a single metabolic biomarker may not provide sufficient diagnostic accuracy. Instead, a metabolic biosignature comprising multiple metabolite classes uniquely associated with TBM may offer a robust and clinically meaningful approach. In this context, free carnitine and acetylcarnitine reflect perturbations in cerebral energy metabolism and mitochondrial dysfunction; tryptophan and quinolinic acid reflect neuroinflammatory processes driven by the tryptophan-kynurenine pathway; and AAs, particularly glycine, which correlates with CSF leucocyte and lymphocyte counts, may provide insight into immune cell activity within the CNS. Although these metabolites were identified using LC-MS, their costs, infrastructure requirements, and technical complexity limit their applicability in routine clinical settings, particularly in resource-constrained environments with the highest TBM burden. However, once validated and clinically relevant thresholds are defined, these candidate metabolites could inform the development of a simplified, low-cost point-of-care assay based on targeted enzymatic, colorimetric, or immunochemical detection. These tests could serve as early indicators of TBM, providing rapid, preliminary diagnostic information that may enable earlier treatment initiation and improved outcomes. A composite metabolic signature may enable more accessible and timely diagnosis of TBM, particularly in resource-limited settings.

## Data Availability

The original contributions presented in the study are included in the article/supplementary material, further inquiries can be directed to the corresponding author.
